# 1-Methyl­piperazine-1,4-diium tetra­chloridozincate hemihydrate

**DOI:** 10.1107/S1600536811043236

**Published:** 2011-10-29

**Authors:** Sondra Walha, Houcine Naïli, Samia Yahyaoui, Tahar Mhiri

**Affiliations:** a Laboratoire de l’Etat Solide, Département de Chimie, Faculté des Sciences de Sfax, BP 1171, 3000 Sfax, Tunisia

## Abstract

The crystal structure of the title compound, (C_5_H_14_N_2_)[ZnCl_4_]·0.5H_2_O, is built up from discrete 1-methyl­piperazine­diium cations with chair conformation, tetrahedral tetrachloridozincate anions and uncoordinated solvent water mol­ecules linked together by three types of inter­molecular hydrogen bonds, *viz.* N—H⋯Cl, N—H⋯O and O—H⋯Cl.

## Related literature

For background on organic–inorganic hybrid materials, see: Lacroix *et al.* (1994 ▶); Mitzi (2001 ▶); Pecaut *et al.* (1993 ▶). For related structures, see: Deeth *et al.* (1984 ▶); Fowkes & Harrison (2004 ▶); Walha *et al.* (2010 ▶, 2011 ▶).
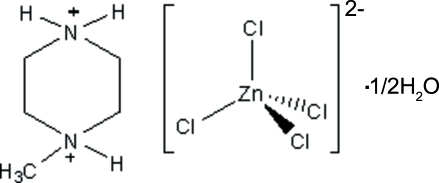


## Experimental

### 

#### Crystal data

(C_5_H_14_N_2_)[ZnCl_4_]·0.5H_2_O
*M* *_r_* = 318.36
Monoclinic, 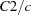

*a* = 14.3210 (5) Å
*b* = 12.7590 (5) Å
*c* = 13.7970 (3) Å
β = 102.821 (3)°
*V* = 2458.16 (14) Å^3^
*Z* = 8
Mo *K*α radiation
μ = 2.83 mm^−1^
*T* = 293 K
0.47 × 0.11 × 0.03 mm

#### Data collection

Nonius KappaCCD diffractometer
Absorption correction: analytical (de Meulenaer & Tompa, 1965 ▶) *T*_min_ = 0.393, *T*_max_ = 0.661
27355 measured reflections
4237 independent reflections
2996 reflections with *I* > 2σ(*I*)

#### Refinement

*R*[*F* ^2^ > 2σ(*F* ^2^)] = 0.050
*wR*(*F* ^2^) = 0.080
*S* = 1.24
4237 reflections
115 parameters
H-atom parameters constrained
Δρ_max_ = 0.49 e Å^−3^
Δρ_min_ = −0.68 e Å^−3^

### 

Data collection: *COLLECT* (Nonius, 1998 ▶); cell refinement: *SCALEPACK* (Otwinowski & Minor, 1997 ▶); data reduction: *DENZO* (Otwinowski & Minor, 1997 ▶) and *SCALEPACK*; program(s) used to solve structure: *SHELXS97* (Sheldrick, 2008 ▶); program(s) used to refine structure: *SHELXL97* (Sheldrick, 2008 ▶); molecular graphics: *DIAMOND* (Brandenburg, 2006 ▶); software used to prepare material for publication: *WinGX* (Farrugia, 1999 ▶).

## Supplementary Material

Crystal structure: contains datablock(s) global, I. DOI: 10.1107/S1600536811043236/zq2122sup1.cif

Structure factors: contains datablock(s) I. DOI: 10.1107/S1600536811043236/zq2122Isup2.hkl

Additional supplementary materials:  crystallographic information; 3D view; checkCIF report

## Figures and Tables

**Table 1:** Selected bond lengths (Å)

Zn—Cl1	2.2449 (8)
Zn—Cl2	2.2614 (7)
Zn—Cl4	2.2615 (8)
Zn—Cl3	2.3004 (7)

**Table 2:** Hydrogen-bond geometry (Å, °)

*D*—H⋯*A*	*D*—H	H⋯*A*	*D*⋯*A*	*D*—H⋯*A*
N1—H1⋯Cl4^i^	0.91	2.31	3.189 (2)	164
N2—H3⋯Cl3^ii^	0.96	2.57	3.353 (2)	139
N2—H3⋯Cl2^ii^	0.96	2.69	3.259 (2)	119
O—H*W*1⋯Cl3	0.96	2.33	3.2692 (12)	167
N2—H2⋯O	0.96	1.95	2.908 (3)	174

Symmetry codes: (i) 
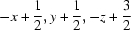
; (ii) 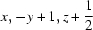.

**Fig. 1.**
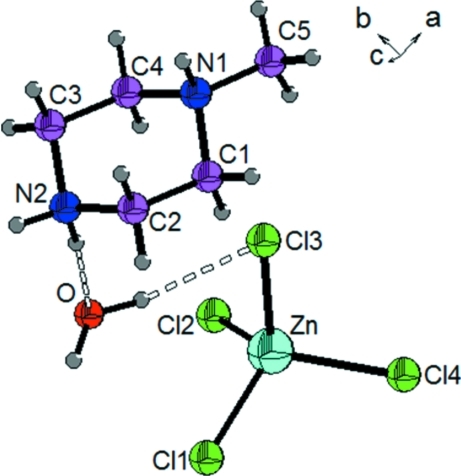
The asymmetric unit of the title compound, with the non-H atoms represented by 50% probability displacement ellipsoids. H atoms are shown as spheres of arbitrary radius.

**Fig. 2.**
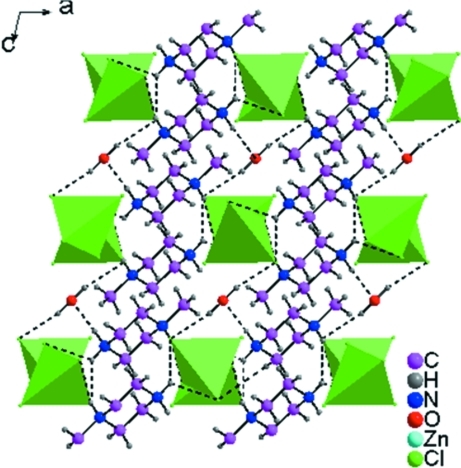
Projection of the crystal structure of the title compound along the b axis, with hydrogen bonds indicated as dashed lines.

**Fig. 3.**
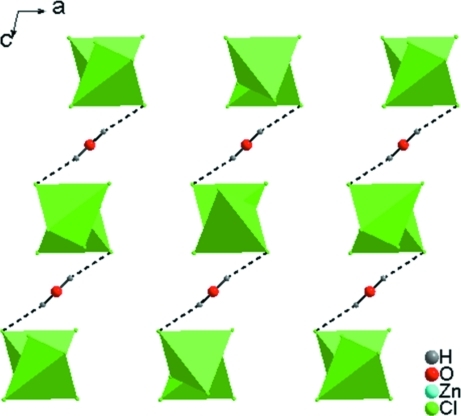
Zig-zag chains formed by the interactions of ZnCl4 and H2O molecules with O—H···Cl hydrogen bonds along the c axis.

## supplementary crystallographic information

### Comment

Preparation of organic-inorganic hybrid materials attracts great attention in chemistry and materials sciences because of their abilities to combine the properties of organic and inorganic compounds within one single molecular scale, such as second-order nonlinear optical (NLO) response, luminescence, magnetism and even multifonctional properties (Mitzi, 2001; Pecaut *et al.*, 1993; Lacroix *et al.*, 1994). In connection with ongoing studies (Walha *et al.*, 2010; Walha *et al.*, 2011), we report here the crystal structure of a new organic-inorganic hybrid with cations and tetrachloridozincate anions.

The asymmetric unit of the title compound (Fig. 1) contains one inorganic ZnCl_4_^2-^ anion, one organic *N*-methylpiperazinediium cation and one half-molecule of water, which lies on a two-fold rotation axis. The isolated molecules form organic-inorganic layers parallels to the (b,c) plane and alternate along the *a* axis (Fig. 2). These layers are stabilized and interconnected by three types of hydrogen bonds: N—H···Cl, N—H···O and O—H···Cl. The anion exhibits a tetrahedral geometry with the Zn^II^ ion surrounded by four Cl atoms with a mean Zn—Cl bond length of 2.267 (2) Å and Cl—Zn—Cl bond angles ranging from 106.24 (3) to 112.42 (3)° (Deeth *et al.*, 1984). The ZnCl_4_ tetrahedra are linked to water molecules into zig-zag chains by O—H···Cl hydrogen bonds along the *c* axis, as illustrated in Fig. 3. The organic species adopts a typical chair conformation with average C—C and C—N of 1.501 (4) and 1.491 (3) Å, respectively (Fowkes & Harrison, 2004). The water molecules are located above and below the layers and they connect them *via* hydrogen bonds. Indeed, they participate in two types of hydrogen bonds O—H···Cl and N—H···O as donor or acceptor, respectively (Table 2), playing a subordinative role in the stabilization of the crystal structure.

### Experimental

ZnCl_2_ (1 mmol) and methylpiperazine dihydrochloride (1 mmol) were dissolved in water. The solution was mixed with hydrochloric acid (1 mmol) and allowed to stand. Colourless plate-shaped crystals of the title compound were formed by slow evaporation of the solvent and separated from the solution after three days.

### Refinement

H atoms bonded to C and N atoms were positioned geometrically and allowed to ride on their parent atom, with C—H = 0.96 Å, N—H = 0.89 Å and *U*_iso_ = 1.2*U*_eq_(C, N).

### Crystal data

**Table:**

(C_5_H_14_N_2_)[ZnCl_4_]·0.5H_2_O	*Z* = 8
*M**_r_* = 318.36	*F*(000) = 1288
Monoclinic, *C*2/*c*	*D*_x_ = 1.720 Mg m^−^^3^
Hall symbol: -C 2yc	Mo *K*α radiation, λ = 0.71073 Å
*a* = 14.3210 (5) Å	θ = 3.5–32.0°
*b* = 12.7590 (5) Å	µ = 2.83 mm^−^^1^
*c* = 13.7970 (3) Å	*T* = 293 K
β = 102.821 (3)°	Plate-shaped, colourless
*V* = 2458.16 (14) Å^3^	0.47 × 0.11 × 0.03 mm

### Data collection

**Table:**

Nonius KappaCCD diffractometer	4237 independent reflections
Radiation source: fine-focus sealed tube	2996 reflections with *I* > 2σ(*I*)
graphite	*R*_int_ = 0.000
Detector resolution: 9 pixels mm^-1^	θ_max_ = 32.0°, θ_min_ = 3.5°
CCD rotation images, thick slices scans	*h* = −21→20
Absorption correction: analytical (de Meulenaer & Tompa, 1965)	*k* = 0→19
*T*_min_ = 0.393, *T*_max_ = 0.661	*l* = 0→20
4237 measured reflections	

### Refinement

**Table:**

Refinement on *F*^2^	Secondary atom site location: difference Fourier map
Least-squares matrix: full	Hydrogen site location: inferred from neighbouring sites
*R*[*F*^2^ > 2σ(*F*^2^)] = 0.050	H-atom parameters constrained
*wR*(*F*^2^) = 0.080	*w* = 1/[σ^2^(*F*_o_^2^) + (0.0157*P*)^2^ + 3.8346*P*] where *P* = (*F*_o_^2^ + 2*F*_c_^2^)/3
*S* = 1.24	(Δ/σ)_max_ < 0.001
4237 reflections	Δρ_max_ = 0.49 e Å^−^^3^
115 parameters	Δρ_min_ = −0.68 e Å^−^^3^
0 restraints	Extinction correction: *SHELXL97* (Sheldrick, 2008)
Primary atom site location: structure-invariant direct methods	Extinction coefficient: 0.0000

### Special details

**Table:**

Geometry. All e.s.d.'s (except the e.s.d. in the dihedral angle between two l.s. planes) are estimated using the full covariance matrix. The cell e.s.d.'s are taken into account individually in the estimation of e.s.d.'s in distances, angles and torsion angles; correlations between e.s.d.'s in cell parameters are only used when they are defined by crystal symmetry. An approximate (isotropic) treatment of cell e.s.d.'s is used for estimating e.s.d.'s involving l.s. planes.
Refinement. Refinement of *F*^2^ against ALL reflections. The weighted *R*-factor *wR* and goodness of fit *S* are based on *F*^2^, conventional *R*-factors *R* are based on *F*, with *F* set to zero for negative *F*^2^. The threshold expression of *F*^2^ > σ(*F*^2^) is used only for calculating *R*-factors(gt) *etc*. and is not relevant to the choice of reflections for refinement. *R*-factors based on *F*^2^ are statistically about twice as large as those based on *F*, and *R*- factors based on ALL data will be even larger.

### Fractional atomic coordinates and isotropic or equivalent isotropic displacement parameters (Å^2^)

**Table:**

	*x*	*y*	*z*	*U*_iso_*/*U*_eq_	
Zn	0.01435 (2)	0.25139 (2)	0.52565 (2)	0.02734 (8)	
Cl1	−0.08951 (5)	0.20675 (6)	0.61886 (5)	0.04259 (18)	
Cl3	0.14474 (5)	0.34080 (6)	0.61526 (5)	0.03835 (16)	
Cl2	−0.05717 (5)	0.35910 (7)	0.40099 (6)	0.0468 (2)	
Cl4	0.07327 (5)	0.11031 (6)	0.45991 (5)	0.04292 (19)	
N2	0.14425 (16)	0.5183 (2)	0.90999 (19)	0.0396 (6)	
H3	0.1119	0.5425	0.9598	0.048*	
H2	0.0992	0.4842	0.8576	0.048*	
N1	0.31874 (14)	0.49919 (18)	0.83935 (15)	0.0288 (5)	
H1	0.3598	0.5316	0.8904	0.035*	
C1	0.27548 (19)	0.4078 (2)	0.8808 (2)	0.0326 (6)	
H1A	0.3258	0.3607	0.9112	0.039*	
H1B	0.2325	0.3726	0.8274	0.039*	
C2	0.2207 (2)	0.4431 (2)	0.9561 (2)	0.0360 (6)	
H2A	0.1925	0.3832	0.9804	0.043*	
H2B	0.2644	0.4764	1.0101	0.043*	
C3	0.1851 (2)	0.6107 (2)	0.8677 (2)	0.0424 (7)	
H3B	0.2258	0.6492	0.9203	0.051*	
H3A	0.1339	0.6550	0.8341	0.051*	
C4	0.2429 (2)	0.5757 (2)	0.7943 (2)	0.0344 (6)	
H4A	0.2004	0.5427	0.7391	0.041*	
H4B	0.2719	0.6359	0.7714	0.041*	
C5	0.3743 (2)	0.4655 (3)	0.7650 (2)	0.0464 (8)	
H5A	0.3369	0.4268	0.7103	0.056*	
H5B	0.4265	0.4215	0.7970	0.056*	
H5C	0.3991	0.5263	0.7382	0.056*	
O	0.0000	0.4275 (2)	0.7500	0.0420 (7)	
HW1	0.0338	0.3954	0.7050	0.050*	

### Atomic displacement parameters (Å^2^)

**Table:**

	*U*^11^	*U*^22^	*U*^33^	*U*^12^	*U*^13^	*U*^23^
Zn	0.02624 (14)	0.02913 (16)	0.02555 (15)	0.00289 (13)	0.00340 (10)	0.00195 (13)
Cl1	0.0478 (4)	0.0476 (4)	0.0363 (4)	−0.0085 (3)	0.0176 (3)	0.0033 (3)
Cl3	0.0348 (3)	0.0437 (4)	0.0338 (4)	−0.0077 (3)	0.0017 (3)	−0.0049 (3)
Cl2	0.0306 (3)	0.0632 (5)	0.0451 (4)	0.0098 (3)	0.0053 (3)	0.0291 (4)
Cl4	0.0473 (4)	0.0451 (4)	0.0321 (4)	0.0176 (3)	−0.0002 (3)	−0.0089 (3)
N2	0.0286 (12)	0.0513 (16)	0.0420 (14)	−0.0053 (11)	0.0141 (10)	−0.0153 (12)
N1	0.0209 (10)	0.0452 (14)	0.0197 (10)	−0.0038 (9)	0.0030 (8)	0.0009 (9)
C1	0.0307 (13)	0.0359 (15)	0.0315 (14)	0.0033 (11)	0.0074 (11)	0.0070 (11)
C2	0.0350 (14)	0.0465 (18)	0.0285 (14)	−0.0083 (13)	0.0117 (11)	0.0023 (12)
C3	0.0401 (16)	0.0379 (17)	0.0486 (18)	0.0067 (13)	0.0088 (13)	−0.0043 (14)
C4	0.0363 (14)	0.0353 (16)	0.0305 (14)	0.0031 (12)	0.0054 (11)	0.0054 (12)
C5	0.0294 (14)	0.081 (2)	0.0313 (15)	0.0083 (15)	0.0129 (12)	0.0041 (15)
O	0.0452 (17)	0.0406 (17)	0.0374 (16)	0.000	0.0033 (13)	0.000

### Geometric parameters (Å, °)

**Table:**

Zn—Cl1	2.2449 (8)	C1—H1A	0.9600
Zn—Cl2	2.2614 (7)	C1—H1B	0.9599
Zn—Cl4	2.2615 (8)	C2—H2A	0.9599
Zn—Cl3	2.3004 (7)	C2—H2B	0.9601
N2—C2	1.488 (4)	C3—C4	1.511 (4)
N2—C3	1.490 (4)	C3—H3B	0.9599
N2—H3	0.9600	C3—H3A	0.9599
N2—H2	0.9599	C4—H4A	0.9601
N1—C4	1.489 (3)	C4—H4B	0.9600
N1—C1	1.492 (3)	C5—H5A	0.9601
N1—C5	1.494 (3)	C5—H5B	0.9599
N1—H1	0.9100	C5—H5C	0.9601
C1—C2	1.503 (4)	O—HW1	0.9600
			
Cl1—Zn—Cl2	110.12 (3)	N2—C2—C1	110.2 (2)
Cl1—Zn—Cl4	112.42 (3)	N2—C2—H2A	109.6
Cl2—Zn—Cl4	108.97 (3)	C1—C2—H2A	109.2
Cl1—Zn—Cl3	112.33 (3)	N2—C2—H2B	109.7
Cl2—Zn—Cl3	106.50 (3)	C1—C2—H2B	108.6
Cl4—Zn—Cl3	106.24 (3)	H2A—C2—H2B	109.5
C2—N2—C3	111.2 (2)	N2—C3—C4	110.5 (2)
C2—N2—H3	109.1	N2—C3—H3B	109.6
C3—N2—H3	108.6	C4—C3—H3B	109.1
C2—N2—H2	109.7	N2—C3—H3A	109.4
C3—N2—H2	108.7	C4—C3—H3A	108.9
H3—N2—H2	109.5	H3B—C3—H3A	109.5
C4—N1—C1	110.2 (2)	N1—C4—C3	111.8 (2)
C4—N1—C5	110.6 (2)	N1—C4—H4A	108.6
C1—N1—C5	111.6 (2)	C3—C4—H4A	108.4
C4—N1—H1	108.1	N1—C4—H4B	109.5
C1—N1—H1	108.1	C3—C4—H4B	109.1
C5—N1—H1	108.1	H4A—C4—H4B	109.5
N1—C1—C2	110.8 (2)	N1—C5—H5A	113.4
N1—C1—H1A	108.8	N1—C5—H5B	109.4
C2—C1—H1A	110.0	H5A—C5—H5B	107.6
N1—C1—H1B	108.7	N1—C5—H5C	109.3
C2—C1—H1B	109.0	H5A—C5—H5C	107.6
H1A—C1—H1B	109.5	H5B—C5—H5C	109.5

### Hydrogen-bond geometry (Å, °)

**Table:**

*D*—H···*A*	*D*—H	H···*A*	*D*···*A*	*D*—H···*A*
N1—H1···Cl4^i^	0.91	2.31	3.189 (2)	164.
N2—H3···Cl3^ii^	0.96	2.57	3.353 (2)	139.
N2—H3···Cl2^ii^	0.96	2.69	3.259 (2)	119.
O—HW1···Cl3	0.96	2.33	3.2692 (12)	167.
N2—H2···O	0.96	1.95	2.908 (3)	174.

Symmetry codes: (i) −*x*+1/2, *y*+1/2, −*z*+3/2; (ii) *x*, −*y*+1, *z*+1/2.
